# The effects of elastic band exercises and nutritional education on frailty, strength, and nutritional intake in elderly women

**DOI:** 10.20463/pan.2020.0007

**Published:** 2020-03-31

**Authors:** Yena Bong, Wook Song

**Affiliations:** 1 Health and Exercise Science Laboratory, Institute of Sports Science, Seoul National University, Seoul Republic of Korea

**Keywords:** Frailty, Elderly women, Elastic band exercise, Nutrition education, Strength, Nutritional intake

## Abstract

**[Purpose]:**

The purpose of this study was to examine the effects of elastic band exercises and nutritional education, as well as to identify the factors influencing frailty, strength, and nutritional intake of elderly women.

**[Methods]:**

The subjects in this study were 30 elderly women who were divided into four groups. All groups agreed to participate in four programs: health education only (HE), elastic band exercises only (EX), nutritional education only (NU), and elastic band exercises plus nutritional education (EX+NU). Frailty was evaluated by measuring the frailty factors according to Fried et al. Leg strength was measured using a leg-extension machine. Nutritional intake was assessed by the 24-hour recall method and food records. Nutritional intake was analyzed by CAN Pro 5.0 program.

**[Results]:**

After three months, the prevalence of frailty significantly decreased in the EX+NU group (P=0.013) compared with that of the HE group (P=0.088). There was significant improvement in leg strength in both the EX (P=0.012) and EX+NU groups (P=0.003) compared with that of the HE group (EX, P=0.005; EX+NU, P=0.002). The nutritional intake significantly decreased in the EX group compared with that of the HE group (P<0.05, P<0.05).

**[Conclusion]:**

The combination of elastic exercises and nutrition education had positive effects on frailty and leg strength, while having negative effects on total calories, carbohydrate, sodium, and iron intake in elderly women. Elastic exercises only had positive effects on leg strength while having negative effects on nutritional intake in elderly women.

## INTRODUCTION

According to the 2017 Elderly Statistics announced by Statistics Korea, 13.8% of the entire population in Korea are senior citizens aged 65 years and above^1^. Moreover, Korea became an aged society in 2018 due to an increased lifespan, improved living standards, and low birth rates, and is expected to become a super-aged society by 2026. This rapidly growing elderly population is becoming a potentially major economic burden on society. According to the World Health Organization (WHO)^2^ and Kurimori et al.^3^ the average lifespan in Korea is 78.6 years, which tends to be high among advanced countries. However, the disability adjusted life expectancy (DALE), which is the time in which one survives without a disability, is 68.6 years, showing a 10-year difference from the average lifespan. This indicates that the elderly’s quality of life in Korea is not desirable due to health, compared to other advanced countries. Moreover, according to Statistics Korea, life expectancy of men and women is 79.3 years and 85.4 years, respectively, indicating that women live longer than men^4^. Statistics Korea also showed that there are more elderly women than men^5^. Sergi et al.^6^ claimed in their cohort study that there are more elderly women in pre-frailty. Therefore, this study focused on elderly women that take up a high percentage of the elderly population.

Along with a growing interest in DALE and successful aging, frailty helps us to understand the diversity of elderly health statuses that are classified as a threat. Defining and classifying frailty helps predict the negative cycle that deteriorates health. Therefore, frailty must be well managed through adequate interventions to prevent it from turning into a disability. According to Mitnitsk et al.^7^ elderly women are especially weaker and showed lower mortality than elderly men at all ages. This is why frailty prevention in elderly women is important.

According to Fried et al.^8^ frailty indicates biological and physiological changes that occur as the general functions decline due to aging, and it is clinically defined as a state in which the decreased physiological function from aging weakens the response system towards external stress, thereby increasing disease morbidity or disability risk.

**Figure 1.**
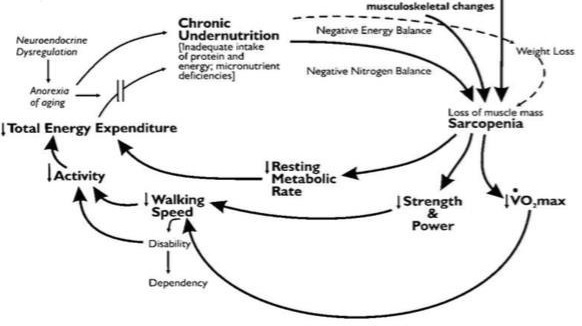
Vicious cycle of frailty (L. P. Fried et al., 2001)^8^.

In the cycle of frailty, decreases in dietary intake due to a loss of appetite causes undernutrition, which results in sarcopenia. This leads to decreased overall physical function as well as fatigue, lack of vitality, and decreased muscular strength, thereby showing a vicious cycle of reduced physical function and walking speed, lack of activity, and decline of energy consumption. In the end, the risk of frailty turning into a disability increases, and makes the elderly more vulnerable to risk factors of detrimental health (such as falls, disabilities, hospitalization, and death) [Fig.1].

Interventions of frailty include nutrition management, hormone replacement therapy, protein and vitamin D treatments, and exercising. Frailty is not something that can be managed completely by a single therapy. Therefore, it is necessary to develop ways to complexly manage frailty using various treatments in order to prevent or delay frailty. In particular, nutritional intake and sarcopenia have a great impact on the cycle of frailty, and protein intake and muscular exercise play a significant role in preventing sarcopenia. Therefore, this study determines how frailty, muscular strength, and nutritional intake change in elderly women by providing them with elastic band exercises and nutritional education.

## METHODS

### Participants

This study selected four welfare centers in Seoul that granted permission to implement the programs after explaining the research purpose and methodology. The participants were 52 elderly women aged 65 years and above who lived in Seoul and volunteered to participate in the study. The participants were limited based on the following selection criteria for the exercise and nutritional education programs: able to communicate; healthy enough to participate; did not receive health education in the last 3 months; did not receive nutritional education in the last 3 months; did not exercise in the last 3 months; can walk 10 m independently; and can move according to directions.

There were 12 individuals that did not meet the selection criteria but 40 individuals passed and participated in the pretest. Ten of them dropped out during the programs for personal reasons or had less than 80% attendance. Ultimately, the data of 30 participants were used in the analysis.

For ethical protection of the participants, approval from the Seoul National University’s Institutional Review Board (IRB) was obtained (IRB No. 1807/003-006).

### Research design

This study is a single blind study (SBS) in which elastic band exercises and nutritional education programs were carried out for 12 weeks with elderly women aged 65 years and above. The participants were divided into the Health Education (HE), Band Exercise (EX), Nutritional Education (NU) group, and Band Exercise + Nutritional Education (EX+NU) groups.

The programs were allocated to 4 selected welfare centers in Seoul that volunteered to participate in the programs. All participants participated after a pretest of body composition, frailty, muscular strength, and nutritional intake.

For 12 weeks, the HE group (N=5) took lectures on overall elderly health, excluding exercise and nutritional education, once a week for an hour. The health program in this study is summarized in Table 1. The EX group (N=9) did elastic band exercises (warm-up and cool-down: 5 minutes each; main exercise: 50 minutes) 3 times a week for an hour. The exercise program in this study is summarized in Table 2. The NU group (N=5) took lectures on elderly related nutritional education once a week for an hour. The nutritional program in this study is summarized in Table 3. The EX+NU group (N=11) did elastic band exercises 3 times a week for an hour and took lectures on elderly related nutritional education once a week for an hour.

**Table 1.** Health program

Week	Content
1	Healthcare in winter
2	Fall prevention education
3	How to prevent and cope with obesity
4	Preventing and managing diabetes
5	Preventing cardiovascular diseases
6	How to prevent and cope with osteoporosis
7	Understanding and managing aging
8	Healthcare for dementia
9	Preventing and managing arthritis
10	Understanding and managing cancer
11	Understanding and preventing depression
12	How to prevent suicide

**Table 2.** Exercise program

	Weeks 1-4	Weeks 5-8	Weeks 9-12
1	Biceps curl (band)	Biceps curl (band)	Biceps curl (band)
2	Triceps extension (band)	Triceps extension (band)	Triceps extension (band)
3	Shoulder press (band)	Open arm (band)	Overhead press (band)
4	Knee push-up	Front raise (band)	Lateral raise (band)
5	Good morning (band)	Step-up with kick back (band)	Step-up with kick back (band)
6	Bodyweight squat	Good morning (band)	Bodyweight lunge
7	Seated row (band)	Bodyweight lunge	Good morning (band)
8	Lower back exercise (band)	Seated row (band)	Squat (band)
9	Bridge	Lower back exercise (band)	Lower back exercise (band)
10	Leg raise	Scissors kick	Scissors kick

**Table 3.** Nutritional program

Week	Content
1	Carbohydrate
2	Protein
3	Fat
4	Vitamin
5	Mineral
6	Aging and nutrition
7	Understanding obesity and weight control
8	Osteoporosis and nutrition management
9	Nutritional management for cancer
10	Nutritional management for diabetes
11	Nutritional management for renal diseases
12	Nutritional management for gastrointestinal diseases

**Figure 2.**
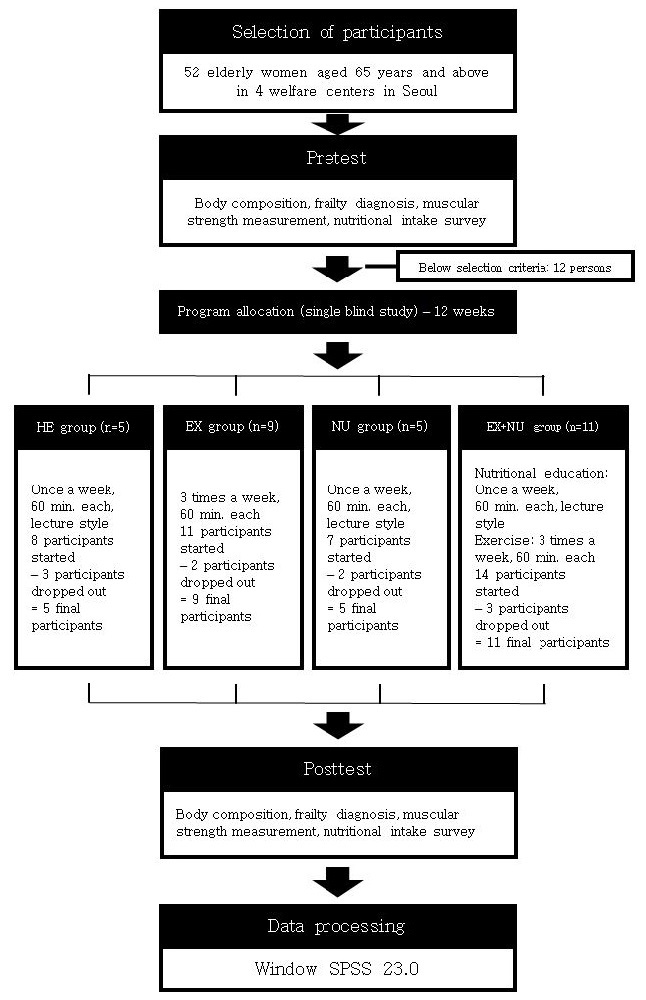
Research design.

The research design is summarized in Figure 2.

### Demographic characteristics and anthropometric data

Data for demographic characteristics were collected through a survey on age, gender, residence, distance to the region where the programs were implemented, means of transport, smoking status, drinking status, years of education, subjective health status, and morbid conditions (diseases).

For body composition, the participants had their height measured using a manual extensometer, and weight, skeletal muscle, body fat, body mass index (BMI), body fat percentage, and waist-hip ratio (WHR) measured with an Inbody 370 (Bio-space, Seoul Korea) using bioelectrical impedance analysis.

For anthropometric data, the same rater measured the thigh circumference halfway between the knee and pelvis. Waist circumference was measured halfway between the bottom of the ribs and top of the iliac crest as the participant looked straight ahead, standing in an upright position with both feet spread shoulder-width apart.

### Frailty

Frailty was rated using five items developed by Fried in a CHS study on the scale of 1-5 (0: Robust; 1-2: pre-frailty; 3-5: frailty) [Table 4].

**Table 4.** Simplified frailty criteria by Fried

Feature	Criteria
Weight loss	Weight loss of at least 5% or 4.5 kg in the past year (except for weight loss due to diet or exercise).
Weakness	When in the bottom 20% of BMI by gender, measured with a digital dynamometer (Men ≤30 kg; Women ≤18 kg).
Exhaustion	Used the Centre for Epidemiological Studies-Depression Scale (CES-D) When the participant answered “frequently (3-4 days)” or “always (5-7 days)” to the two questions, “How often did you feel that everything you did was an effort?” and “How often did you feel that you could not get going?” in the past week.
Slowness	When it takes at least 0.8/sec to walk 4 m without an assist device.
Low activity	When low according to the International Physical Activity Questionnaire (IPAQ); short form).

### Muscular strength

Grip strength represents arm length and was measured by having the participant lift both arms slightly while standing upright with both feet spread shoulder-width apart. Two measurements were taken by turns for both the left and right using a digital dynamometer (Takei Scientific Instruments Co., Niigata, Japan), and the means were used in the analysis.

Leg strength was measured using a leg strength instrument (to measure isometric knee extensor force, T.K.K 5710M, Japan). The participant sat on the instrument, which was adjusted around the legs and set at two-thirds from the top of the front shin. The participant leaned back completely and put on a waist belt, setting the instrument to 0 by pressing the button, and the participant then pushed the instrument upward with all her strength. The maximum value measured was used in the analysis.

### Nutritional intake

Nutritional intake of the participants was examined using a 24-hour diet recall through individual interviews with trained dietitians. Intake of each nutrient was analyzed using the Computer Aided Nutritional Analysis Program (CAN-Pro 5.0) developed by the Korean Nutrition Society.

### Health education program

The health education program was newly formed by trained health specialists based on all kinds of data. The details are presented in Table 1.

### Exercise program

The exercise program was a revised version of the High-Speed Band-Based Power Training (HSPT) in a study conducted by the Seoul Metropolitan Government. The rating of perceived exertion was used before the exercise to set the band strength; and accordingly, the bands mainly used were yellow, which is the weakest, and red, which is one level stronger. Exercise intensity was set at 12-13 based on the rating of perceived exertion. For 12 weeks, the participants performed warm-ups, cool-downs, and main exercises 3 times a week for an hour. The exercise intensity was gradually increased by dividing the program into three sessions. The intensity was increased by adjusting the strength or length of the band after a month. The details are presented in Table 2.

### Nutritional education program

The nutritional program was reconstructed by professional dietitians with reference to the elderly dietitian training program of the Korean Dietetic Association. The details are presented in Table 3.

### Statistical analysis

All data measured in this study were analyzed using Windows SPSS 23.0. The means and standard deviations of variables were calculated using descriptive statistics.

A Shapiro-Wilk test was conducted to test the normality of data, and the results showed that some variables were not normally distributed, and since the number of samples was small, non-parametric statistics were used. A Kruskal-Wallis test was conducted to test the homogeneity of the four groups. A Wilcoxon signed-rank test was conducted to compare the pretest and posttest results within a group, and a Kruskal-Wallis test was used to compare the variance due to interventions among the groups. When there was a level difference, a Wilcoxon rank sum test was used to pair the groups and analyze which ones showed a level difference.

## RESULTS

### Changes in body composition

Changes in body composition of the four groups after 12 weeks of elastic band exercises and nutritional education are presented in Table 5. Among the items of body composition in the four groups before and after the programs, changes in waist circumference were statistically significant (p<0.05). Other variables were not statistically significant (p>0.05). In the EX+NU group, changes in weight (p<0.1) and BMI (p<0.05) according to time were significant. In the HE and NU groups, changes in waist circumference according to time were significant (p<0.05).

**Table 5.** Changes in body composition

Item	Group	Pretest	Posttest	Time	Time*Group
Weight (kg)	HE group EX group NU group EX+NU group	63.48 ± 10.06 56.67 ± 6.65 50.84 ± 2.4 54.16 ± 8.24	63.56 ± 9.9 56.56 ± 6.06 50.3 ± 2.03 53.22 ± 7.18	0.786 0.624 0.225 0.053	0.494
Skeletal muscle (kg)	HE group EX group NU group EX+NU group	23.58 ± 7.62 20.16 ± 1.55 17.24 ± 1.39 19.48 ± 2.13	21.26 ± 1.98 20.17 ± 1.38 16.7 ± 3.18 19.1 ± 1.77	0.893 0.833 1 0.333	0.842
Body fat (kg)	HE group EX group NU group EX+NU group	32.28 ± 11.06 18.95 ± 4.5 17.68 ± 2.95 31.56 ± 7.44	23.86 ± 7.38 18.92 ± 5.68 18.14 ± 4.28 17.19 ± 6.04	0.686 0.514 0.5 0.689	0.893
BMI (kg/m^2^)	HE group EX group NU group EX+NU group	27.7 ± 3.44 23.48 ± 2.51 22.42 ± 1.57 23.59 ± 3.13	27.36 ± 4.22 23.44 ± 2.24 22.14 ± 1.25 23.16 ± 2.77	0.686 0.798 0.176 0.036*	0.617
Body fat percentage (%)	HE group EX group NU group EX+NU group	32.28 ± 11.06 32.98 ± 4.63 34.68 ± 4.89 31.56 ± 7.44	36.76 ± 7.33 32.88 ± 7.09 36.24 ± 9.71 31.55 ± 7.97	0.686 0.767 0.5 0.859	0.946
Abdominal fat ratio	HE group EX group NU group EX+NU group	0.85 ± 0.05 0.85 ± 0.04 0.89 ± 0.02 0.84 ± 0.07	0.88 ± 0.04 0.86 ± 0.05 0.86 ± 0.04 0.84 ± 0.07	0.715 0.105 0.221 0.778	0.199
Thigh circumference (cm)	HE group EX group NU group EX+NU group	50.2 ± 5.46 46.34 ± 10.41 43.88 ± 0.58 47.45 ± 4.35	48.8 ± 3.85 48.8 ± 3.15 43.5 ± 0.86 51.09 ± 13.9	0.345 0.262 0.465 0.965	0.5
Waist circumference (cm)	HE group EX group NU group EX+NU group	99.2 ± 10.63 86.06 ± 6.2 88.82 ± 4.08 83.64 ± 10.01	94.6 ± 10.3 85.5 ± 6.24 82.9 ± 5.77 79.75 ± 14.4	0.043* 0.594 0.043* 0.965	0.008*

Values are the mean ± standard deviation, P<0.05(*) Pre vs. Post

### Changes in the frailty state

**Figure 3.**
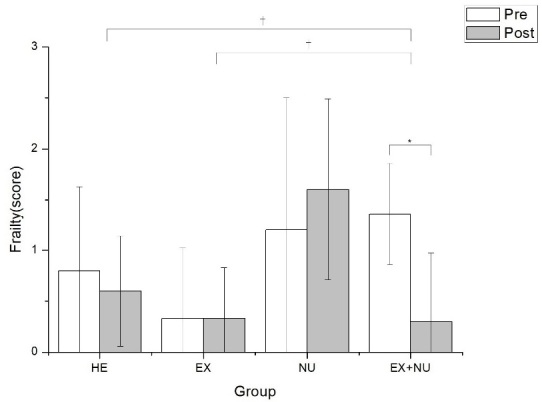
Changes in the frailty state (Score). Values are the mean ± standard deviation, P<0.05(*) Pre vs. Post, p<.05(†) vs. HE

Changes in the frailty state of the four groups after 12 weeks of elastic band exercises and nutritional education are presented in Table 6. Changes in frailty of the four groups after the programs were statistically significant (p<0.05). Changes according to time were significant in the EX+NU group (p<0.05). For changes in the frailty state of the EX+NU group, changes in walking speed according to time were significant (p<0.05; Table 7; Fig. 3).

**Table 6.** Changes in the frailty state

Group	Pretest	Posttest	Time	Time*Group
HE group	0.8 ± 0.83	0.6 ± 0.54	0.564	0.047*
EX group	0.33 ± 0.7	0.33 ± 0.5	1	
NU group	1.2 ± 1.3	1.6 ± 0.89	0.577	
EX+NU group	1.36 ± 0.5	0.36 ± 0.67	0.013*	

Values are the mean ± standard deviation, P<0.05(*) Pre vs. Post

**Table 7.** Changes in the frailty state of the EX+NU group

Frailty	Pretest	Posttest	Time
Weight loss	0	0	1
Slowness	0.72 ± 0.46	0.18 ± 0.4	0.034*
Weakness	0	0.18 ± 0.4	0.157
Exhaustion	0.18 ± 0.4	0	0.157
Low activity	0.27 ± 0.46	0.09 ± 0.3	0.317

Values are the mean ± standard deviation, P<0.05(*) Pre vs. Post

### Changes in muscular strength

Changes in muscular strength of the four groups after 12 weeks of elastic band exercises and nutritional education are presented in Table 8. Changes in grip strength of the four groups after the programs were not statistically significant (p>0.05) but changes in leg strength were statistically significant (p<0.05). Changes in grip strength according to time were not significant (p>0.05), while changes in leg strength were statistically significant in the HE (p<0.1), EX, and EX+NU groups (p<0.05).

**Table 8.** Changes in muscular strength

Item	Group	Pretest	Posttest	Time	Time*Group
Grip strength (kg)	HE group EX group NU group EX+NU group	22.04 ± 3.14 22.76 ± 4 21.32 ± 2.4 22.26 ± 3.91	21.16 ± 3.08 23.53 ± 3.25 20.88 ± 2.46 23.24 ± 3.8	0.225 0.161 0.686 0.306	0.316
Leg strength (kg)	HE group EX group NU group EX+NU group	53.94 ± 10.02 44.77 ± 13.8 27.68 ± 3.67 36.31 ± 10.88	45.6 ± 4.15 51.6 ± 12.7 34.04 ± 12 46.18 ± 9.43	0.08 0.012* 0.225 0.003*	0.012*

Values are the mean ± standard deviation, P<0.05(*) Pre vs. Post

### Changes in leg strength

**Figure 4.**
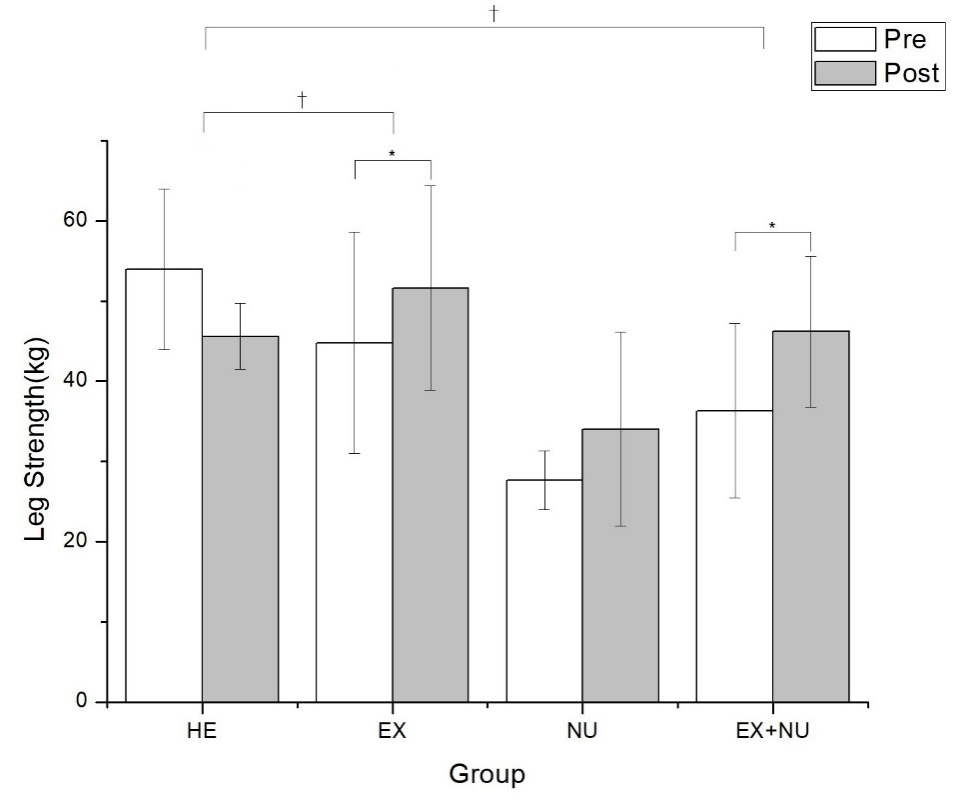
Changes in leg strength (kg). Values are the mean ± standard deviation, P<0.05(*) Pre vs. Post, p<.05(†) vs. HE

Changes in leg strength of the four groups after 12 weeks of elastic band exercises and nutritional education are summarized in Table 9 and Fig. 4.

**Table 9.** Results of leg strength (kg) changes

Group	Pretest	Posttest	Time	Time*Group
HE group	53.94 ± 10.02	45.6 ± 4.15	0.08	0.012*
EX group	44.77 ± 13.8	51.6 ± 12.7	0.012*	
NU group	27.68 ± 3.67	34.04 ± 12	0.225	
EX+NU group	36.31 ± 10.88	46.18 ± 9.43	0.003*	

Values are the mean ± standard deviation, P<0.05(*) Pre vs. Post

Changes in leg strength of the four groups after the programs were statistically significant (p<0.05), and changes according to time were statistically significant in the HE (p<0.1), EX, and EX+NU groups (p<0.05).

### Changes in nutritional intake

Changes in nutritional intake of the four groups after 12 weeks of elastic band exercises and nutritional education are presented in Table 10. Changes in nutritional intake of the four groups after the programs were statistically significant (fat: p<0.1; other variables: p<0.05). Changes according to time were statistically significant in all variables of nutritional intake for the EX group (p<0.05), and only in energy intake for the NU group (p<0.05). For the EX+NU group, changes according to time were statistically significant in energy, carbohydrate, sodium, and iron intake (p<0.05).

**Table 10.** Changes in nutritional intake

Item	Group	Pretest	Posttest	Time	Time*Group
Energy(Kcal)	HE group EX group NU group EX+NU group	1194 ± 257.17 2931.5 ± 464.5 1054.5 ± 281 2603.5 ± 841.2	1146.7 ± 853.5 1268.5 ± 282.2 753.98 ± 355.2 1774.8 ± 340.7	0.893 0.008* 0.043* 0.01*	0.002*
Carbohydrate(g)	HE group EX group NU group EX+NU group	210.02±40.26 460.52±59.92 169.65±53.8 409.71±122.2	190.05 ± 51.86 204.21 ± 52.55 138.54 ± 62.24 244.31 ± 39.5	0.225 0.008* 0.138 0.006*	0.001*
Protein (g)	HE group EX group NU group EX+NU group	42.02 ± 14.49 109.5 ± 26.32 33.16 ± 7.96 94.44 ± 38.14	53.91 ± 39.11 48.28 ± 8.17 22.48 ± 14.81 72.11 ± 17.3	0.686 0.008* 0.225 0.11	0.002*
Fat (g)	HE group EX group NU group EX+NU group	21.36 ± 19.03 75.69 ± 32.19 28.24 ± 12.65 69.04 ± 34.08	37.31 ± 33.13 28.78 ± 8.11 11.59 ± 13.8 59.53 ± 29.7	0.345 0.011* 0.043* 0.328	0.055
Calcium (mg)	HE group EX group NU group EX+NU group	338.95 ± 77.21 839.37 ± 280.8 197.73 ± 54.44 740.71 ± 340.1	395.34 ± 315.8 401.6 ± 196.43 173.8 ± 179.13 587.69 ± 212	0.5 0.011* 0.5 0.155	0.023*
Sodium (mg)	HE group EX group NU group EX+NU group	2039.5 ± 732.1 5506.4 ± 1687 1592 ± 675.71 4665.5 ± 2266	3122 ± 1502.6 2793.7 ± 522.5 1294 ± 1103.6 3228.7 ± 1135	0.225 0.008* 0.5 0.033*	0.008*
Iron (mg)	HE group EX group NU group EX+NU group	10.9 ± 1.89 26.53 ± 4.86 7.37 ± 2.68 23.13 ± 7.28	16.17 ± 9.85 15.38 ± 6.97 5.52 ± 2.67 14.05 ± 4.39	0.225 0.008* 0.225 0.008*	0.002*

Values are the mean ± standard deviation, P<0.05(*) Pre vs. Post

## DISCUSSION

This study aimed to determine the effects of 12 weeks of elastic band exercises and nutritional education on the frailty state, muscular strength, and nutritional intake of elderly women. The results of this study are to be discussed in comparison with previous studies.

### Changes in the frailty state

This study observed the changes in the frailty state of elderly women after 12 weeks of elastic band exercises and nutritional education.

Lipsitz^9^, Lipsitz and Goldberger^10^, and Fried et al.^8^ conceptually defined frailty as a clinical condition that appears in association with aging, in which there is higher vulnerability and decreased physiological capability and organizational systems, and is a state in which the immune reactions are not properly displayed due to daily or temporary stress. Frailty has a vicious cycle in which the loss of appetite reduces dietary intake as well as weight and muscle mass, strength, and power, thereby deteriorating physical functions such as walking speed and physical activity, ultimately resulting in death.

According to Hawkins, Wiswell, and Marcell^11^, the elderly show a decrease in muscular strength and mass as they age, and thus their functional skills also decrease. At least a 30% decrease occurs between the age of 50 and 70 years, after which muscular strength decreases even more rapidly. According to Ferrucci et al.^12^, exercise intervention prevents 5 items of the frailty diagnosis criteria, slows the progression, and helps the elderly return to their pre-frailty status. However, no changes in the frailty state were found in this study when only exercise treatment was used.

Manal^13^ argues that studies on nutritional treatment interventions for frailty are gradually increasing, but the effects are still unclear. According to Daniels^14^, there is insufficient evidence regarding whether nutritional interventions for the elderly with frailty in the community can prevent physical disability in daily living performance.

Milne^15^ claimed that implementing protein and energy treatment for the elderly who are malnourished has a somewhat positive effect on weight gain and mortality reduction, but there is insufficient evidence about the effects on functional improvements. According to Nykänen^16^, providing individual nutritional education for a year to the elderly in frailty or pre-frailty aged 75 years and above improved their frailty state. However, no changes in the frailty state were found in this study when only nutritional education was used.

There was a statistically significant decrease in this study when both exercise and nutritional education were provided (p<.05), which is similar to the results by Seino et al.^17^ that provided 12 weeks of elastic exercises and nutritional and psychosocial programs for the elderly in frailty or pre-frailty^17^.

### Changes in muscular strength

This study observed the changes in the muscular strength of elderly women after 12 weeks of elastic band exercises and nutritional education.

Muscular strength refers to the strength of muscles formed by muscle contraction. This tends to increase until age 30, before slightly weakening during middle age, and then decreases in senescence. However, Choi^18^ claimed that regular exercise can increase muscular strength by 30-50%, and reduce the amount of decrease in senescence. Lee^19^ argues that the aging process of the human body begins at age 40. Muscle mass decreases up to 20-40% between the ages of 20 and 65; the number and size of muscular fibers decreases by 50% between 60-70 years compared to youths aged 21 years, with functional decline of at least 30-50% compared to those in their 30s.

It is known that exercising improves muscular strength. Studies proving the effects of exercising on muscular strength include one by Bullani et al.^20^ which had elderly patients (mean age: 70 years) do leg exercises twice a week for 40 minutes for a total of 18 weeks using an elastic band. The results showed that there was a significant improvement in the Tinetti (gait and balance) and Timed Up and Go tests (physical functions).

SunWoo et al.^21^ revised and improved the Kochi health center elderly exercise program, which revised and improved upon the muscular strength training program for the elderly co-developed by the US National Institute on Aging, US Centers for Disease Control and Prevention, and experts at Tufts University to better fit the Japanese population. The exercise program was modified accordingly with the characteristics of the frail elderly in Korea. As a result of implementing the exercise program on 401 frail elders for 3 months, twice a week for an hour each, the elders showed improvements in grip strength and standing on one leg.

For studies using bands, Kim et al.^22^ implemented elastic band exercises to 14 elderly women aged 60 years and above once a week, 50 minutes each for 15 weeks, and as a result, their grip strength significantly increased. Moreover, Lee et al.^23^ implemented elastic band exercises to 30 elderly women 3 times a week, 50 minutes each for 8 weeks, and the results showed that both arm and leg strength increased.

This study also showed a significant increase in leg strength as a result of exercise treatment (p<.05).

### Changes in nutritional intake

This study observed the changes in the nutritional intake of elderly women after 12 weeks of elastic band exercises and nutritional education.

According to Hong et al.^24^, nutritional intake decreases as people age, which is due to changes in not only taste but also social factors such as changes in home environment, decline of respect for the elderly, nuclearization of families, and increases in double-income couples. These changes result in an increasing number of elders exposed to nutritional risks. Studies on the elderly in Korea generally show a lack of nutrition and a hierarchical gap.

Schlenker^25^ and Splett^26^ argue that the elderly in nutritional risk have trouble moving their bodies, frequently skip meals, cannot afford to spend on food, must eat alone, receive free meals at a soup kitchen, and only have a hot meal once a day. Davies^27^ claim that people are likely to be malnourished when there is nothing to eat at home or when there are environmental factors such as loneliness or depression that causes a loss of appetite. According to Hong et al.^24^, elders are nutritionally vulnerable and have chronic diseases due to physical changes as well as psychological, social, and economic factors. Moreover, Splett^26^ argues that elders have difficulty buying, cooking, or chewing food. Half of the elders in the US are estimated to be suffering from innutrition.

Studies in which nutritional intake changed due to nutritional education are as follows. First, Choi et al.^18^ provided 1-hour nutritional education for 5 weeks to 78 elders. It was found that both the group that completed the program and the group that did not showed insufficient intake of energy (less than 75% of the recommended intake) before and after the program. There was no significant difference in vitamins and minerals as well. However, intake of sodium decreased greatly in the group that completed the program. As such, this study also showed that only energy intake decreased in the group that only received nutritional education, but there were no other changes in nutritional intake. This shows that nutritional education alone is not enough to change the nutritional intake. Moreover, there may have been difficulty due to the fact that there was only nutritional education in this study without controlling for the actual dietary intake.

Nutritional intake showed a statistically significant decrease in the group that received exercise treatment, which was related to appetite. According to Haspolat et al.^28^ leptin, which is an anti-obesity hormone synthesized and secreted from fatty tissues, is highly associated with fat mass, increased energy consumption, and weight control. According to Wang et al.^29^ ghrelin, an antagonist to leptin, is a protein secreted from the cells of the gastric mucosa and affects the hypothalamus. Ghrelin is known to serve as a catalyst for growth hormone release and have an orexigenic effect. King^30^ observed that brief, acute exercises resulted in a rapid decrease in hunger and food intake. As such, exercising increases leptin, which minimizes appetite and thereby reduces nutritional intake.

## CONCLUSION

This study verified the changes in frailty, muscular strength, and nutritional intake of elderly women after 12 weeks of elastic band exercises and nutritional education. First, a mixed treatment of exercise and nutritional education reduced the frailty state with statistical significance. Second, exercise treatment increased leg strength and reduced nutritional intake with statistical significance. According to previous studies, separate treatments of exercise and nutritional education can improve the frailty state, but as proven in the results of this study, a mixed treatment of exercise and nutritional education is more effective in improving the frailty state than a single treatment. Therefore, it is necessary to examine changes in the frailty state by applying various intensities, periods, and improved contents of education in follow-up research.
